# A New Stepwise Molecular Work-Up After Chorionic Villi Sampling in Women With an Early Pregnancy Loss

**DOI:** 10.3389/fgene.2020.561720

**Published:** 2021-01-14

**Authors:** Montse Pauta, Cèlia Badenas, Laia Rodriguez-Revenga, Anna Soler, Maribel Grande, Joan Sabrià, Carmen Illanes, Virginia Borobio, Antoni Borrell

**Affiliations:** ^1^BCNatal, Institut d’Investigacions Biomèdiques August Pi i Sunyer (IDIBAPS), Barcelona, Spain; ^2^Servei de Bioquímica i Genètica Molecular, CDB, Hospital Clínic de Barcelona, Barcelona, Spain; ^3^BCNatal, Servei de Ginecologia i Obstetricia, Hospital de Sant Joan de Déu, Esplugues de Llobregat, Spain; ^4^BCNatal Department of Maternal-Fetal Medicine, Institute Gynecology, Obstetrics and Neonatology, Hospital Clínic de Barcelona, Barcelona, Spain

**Keywords:** prenatal diagnosis, early pregnancy loss, products of conception, chromosomal anomalies, copy number variant, complete hydatidiform mole, chorionic villi sampling

## Abstract

**Objective:** To explore the use of a new molecular work-up based on the stepwise use of Quantitative Fluorescence PCR (QF-PCR) extended to eight chromosomes and single nucleotide polymorphism array (SNP-array) in chorionic villi obtained by chorionic villi sampling (CVS) offered to women experiencing an early pregnancy loss.

**Methods:** During a 3-year period (January 2016–December 2018), CVS was offered to women experiencing an early pregnancy loss before the evacuation of the products of conception (POC) to retrieve chorionic villi, irrespective of the number of previous losses. A new molecular work-up was prospectively assayed encompassing a first QF-PCR round (with the 21, 18, 13, 7, X, and Y chromosomes), a second QF-PCR round (with the 15, 16, and 22 chromosomes), and a high resolution SNP-array in those cases with normal QF-PCR results. A control group in which POC were collected after surgical uterine evacuation was used to be compared with the intervention group.

**Results:** Around 459 women were enrolled in the intervention group (CVS) and 185 in the control group (POC after uterine evacuation). The QF-PCR testing success rates were significantly higher in the intervention group (98.5%: 452/459) as compared to the control group (74%: 109/147; *p* < 0.001), while the chromosomal anomaly rate at the two QF-PCR rounds was similar between the two groups: 52% (234/452) in the intervention and 42% (46/109) in the control group (*p* = 0.073). The SNP-array was performed in 202 QF-PCR normal samples of the intervention group and revealed 67 (33%) atypical chromosomal anomalies (>10 Mb), 5 (2.5%) submicroscopic pathogenic copy number variants, and 2 (1%) variant of uncertain significance (VOUS).

**Conclusion:** Eighty-two percent of women experiencing an early pregnancy loss opted for a CVS. The testing success rates were higher in the intervention group (CVS; 98%) as compared to the control group (POC; 74%). The overall yields were 52% by QF-PCR (including three complete hydatiform moles), and 16% by SNP-array, including 15% atypical chromosomal anomalies and 1.1% submicroscopic pathogenic copy number variants.

## Introduction

Early pregnancy loss is defined as a non-viable, intrauterine pregnancy in the first trimester with either an empty gestational sac or a gestational sac containing an embryo or a fetus without fetal heart activity (Nice.org.uk, 2012). This is the most common form of pregnancy loss, occurring in 15–20% of clinical pregnancies (Zinaman et al., 1996). It is clearly associated with maternal age, increasing from 10% of pregnancies at 20 years to 75% at 40 years (Andersen et al., 2000) due to a rise in chromosomally abnormal pregnancies along maternal age (Grande et al., 2012). Our group has recently shown that 70% of first trimester miscarriages are associated with chromosomal abnormalities, with autosomal trisomies being the most common type (65%), followed by triploidies (13%), monosomies X (10%), and structural rearrangements (5%; Soler et al., 2017).

In order to diagnose chromosomal abnormalities in recurrent losses, G-Banding karyotyping in products of conception (POC) has traditionally been used with a limited success rate. The quality and viability of POC samples are often suboptimal, resulting in a 32% culture failure rate according to a recent meta-analysis (Pauta et al., 2018). In addition, maternal cell-contamination leads to a false-negative result in 22% of the samples (Lathi et al., 2014) decreasing to 53% of the rate of correct diagnoses in POC samples.

In this study, we address early pregnancy loss from three novel approaches: (a) We performed chorionic villi sampling (CVS) with a transcervical forceps before uterine evacuation; (b) we applied a new stepwise molecular work-up based on the use of Quantitative Fluorescence PCR (QF-PCR) extended to eight chromosomes and single nucleotide polymorphism array (SNP-array); and (c) we offered this work-up to women after their first early pregnancy loss, since a single early gestational loss has been shown to have a great emotional impact (Stergiotou et al., 2016; Farren et al., 2018).

## Materials and Methods

This is an interventional trial in which two rounds of QF-PCR (first step) and SNP-array (second step) were applied to chorionic villi retrieved by CVS before uterine evacuation of POC. This trial was developed in women who experienced a pregnancy loss before week 14 to diagnose genetic anomalies causing that loss. This study was approved by IRB of Hospital Clinic de Barcelona (HCB/2014/6020), and written informed consent was obtained from all women.

### Study Population

Consecutive women with an early pregnancy loss were enrolled in the two BCNatal sites, either to the intervention group in HCB during a 3-year period (January 2016–December 2018) or to the control group in Hospital Sant Joan de Déu (HSJD) during a 2-year period (January 2016–December 2017). The intervention group underwent a CVS before either a medical or a surgical uterine evacuation, while POC in the control group were obtained after surgical evacuation. Women were offered to participate in the study when an early pregnancy loss was diagnosed. Participation in the study was open to women referred to the Prenatal Diagnosis Unit because a non-viable pregnancy was detected either in: (a) the Ultrasound Department during the 10–13 weeks’ scan; (b) the Emergency Department after consultation of women with bleeding or cramps; or (c) the Reproduction Department. An informed consent was obtained from all mothers. This document provided the choice of not being informed of possible secondary findings.

### Ultrasound Examination

Ultrasound examination was performed with the use of a transvaginal probe and included the measurement of gestational sac diameters or the crown-rump length if an embryo was present. Examinations were performed using the Acuson Antares system (Siemens Medical Solutions, Malver, PA, United States) or Voluson E8 (GE Medical System, Zipf, Austria).

### Sample Collection

In both study groups, samples were collected in warmed RPMI 1640 medium (BioWhittaker, Cambrex, Belgium) and delivered to the laboratory within a few hours. These samples were evaluated and processed as described elsewhere (Morales et al., 2008; Stergiotou et al., 2016). Genomic DNA was isolated from chorionic villus cells or POC using the Qiagen Mini Kit (Qiagen, Valencia, CA) following the manufacturer’s protocol. DNA concentration and quality were measured with Qubit 2.0 fluorometer (Life Technologies Inc.) and NanoDrop 200c spectrophotometer (Thermo Fisher Scientific Inc.).

### Quantitative Fluorescent PCR

Two rounds of QF-PCR were performed as the first step of analysis. The first round encompassed the chromosomes 13, 18, 21, X, and Y. In addition to detecting trisomy 13, 18, and 21 and sex aneuploidies, this technique is able to detect triploidies, complete androgenetic uniparental disomies (that cause the complete hydatiform mole), and maternal cell contamination (if a maternal saliva sample was taken; Badenas et al., 2010). In case of a normal result, a second QF-PCR round was carried out to assess the chromosomes 15, 16, and 22. The first QF-PCR round, Devyser Compact v3 QF-PCR kit, amplifies simultaneously 26 markers of the five studied chromosomes, while Devyser Extend v2 amplifies 15 markers from chromosomes 16, 18, and 22 in the second round. The PCR products were run on an ABI3130XL Genetic Analyzer (ABI, Foster City, CA, United States), and results were analyzed with GeneMapper 4 software (ABI). Significant maternal contamination was ruled out with the use of microsatellite markers for chromosomes 13, 18, and 21 included in the QF-PCR kit.

### Single Nucleotide Polymorphism-Array

Chromosomal Microarray Analysis (CMA) was carried out using an SNP-array (SurePrint G3 Custom CGH+SNP, 4x180K; Agilent, Santa Clara, CA, United States) that allows simultaneous detection of copy number variations (CNV) and copy neutral aberrations, such as loss of heterozygosity (LOH) and uniparental disomy (UPD). This array targets ∼500 ISCA regions described in the International Standards for Cytogenomics Array consortium and has a 25 Kb backbone probe density and a 5–10 Mb LOH/UPD resolution. The slides were scanned on an Agilent G2565CA Microarray Scanner System (Agilent Technologies, Santa Clara, CA, United States), and images were analyzed using Cytogenomics software (version 5.0, Agilent Technologies, Santa Clara, CA, United States). Results were presented on the human genome assembly hg19.

### Classification of Variants

Pathogenicity of variants was assessed taking into account its previous classification in Online Mendelian Inheritance in Man (OMIM), human genome browsers (UCSC, Ensembl), Database of Chromosomal Imbalance and Phenotype in Humans using Ensembl Resources (DECIPHER) databases, or its presence in general population datasets [Database of Genomic Variants (DGV)]. Variants were classified according to the American College of Medical Genetics and Genomics (ACMG) guidelines (Riggs et al., 2020).

### G-Banding Karyotyping

The new work-up was assayed concurrently with our routine protocol that includes two karyotypes, one in short-term and one in long-term culture. The short-term culture allows to avoiding maternal cell contamination, given that the origin of any 46,XX cell line must be of cytotrophoblast origin, the only cell source with spontaneous mitoses. The karyotype was assessed in 20 metaphases from the short-term culture and five from the long-term culture, according to European Cytogenetics Association guidelines (Hastings et al., 2007). G-banding was performed following the standard protocol according to the International System for Human Cytogenetic Nomenclature 2016 and using the Wright technique (International Standing Committee on Human Cytogenomic Nomenclature, 2016).

### Management

Women in the intervention group were able to choose whether they prefer expectant, medical (when the embryo’s crown-rump length was shorter than 23 mm), or surgical, management, while in the control group all women had surgical management, since medical management was not available at HSJD. CVS was not performed in the operation room at the time of surgery. After CVS, women were given an appointment for genetic counseling 4 weeks after the procedure to discuss the anomaly detected and its recurrence risk (Grande et al., 2017).

### Data Entry and Statistical Analysis

Data on maternal characteristics and pregnancy ultrasound features were registered in a Statistical Package for the Social Science (SPSS) database (SPSS, Chicago, Illinois, United States), which was then used for statistical analyses. The Kolmogorov-Smirnov test was used to assess whether variables were normally distributed. For variables not normally distributed, the Mann-Whitney test was used to determine significant differences between groups. Chi-square statistics and the Fisher exact test were used to examine differences between proportions. Means and SD were used for normally distributed variables and the *t*-test for comparisons. A two-sided value of *p* < 0.05 was considered as statistically significant.

## Results

Among women with a pregnancy loss up to 14 weeks of gestation attending HCB during the study period (2016–2018), 459 were enrolled in the intervention group. HSJD enrolled 185 women as controls (2016–2017). The median menstrual gestational age at pregnancy loss was 9.7 weeks (range 5–13 weeks) in the intervention group and 10 weeks (range 5–13) in the control group. The median sonographic gestational ages were 8 + 1 weeks (range 5–13 weeks) and 6 + 5 weeks (range: 5–12 weeks). When no embryo was observed, 5 weeks were assigned as the estimated sonographic gestational age. Maternal tissue was the only content of five (1%) samples of the intervention group and of 38 (26%) of the control group and were excluded from further testing, leaving 454 and 147 cases in each group, respectively.

**Figure 1:**
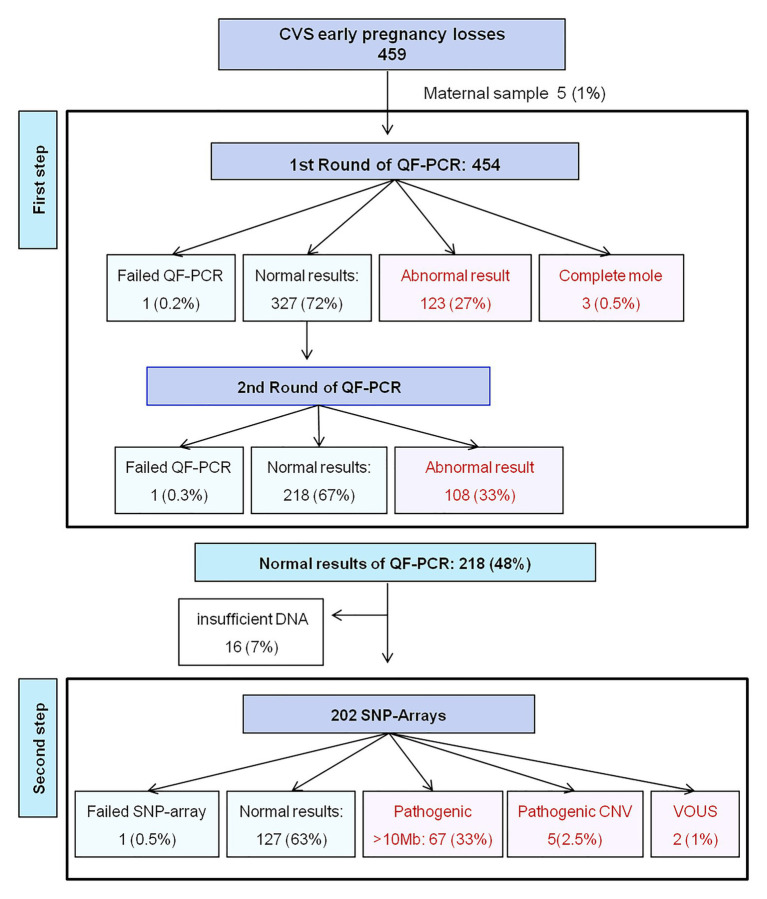
Flow diagram of the intervention group in which the stepwise work-up was applied. CVS, Chorionic villi sampling; QF-PCR, Quantitative Fluorescent PCR; SNP, Single Nucleotide Polymorphism; CNV, Copy Number Variations; VOUS, Variant of Uncertain Significance.

As the first testing step, 454 chorionic villi samples of the intervention group (Figure 1) and 147 of the control group were assessed by two sequential QF-PCR rounds. The testing success rates were significantly different between the intervention (98.5%: 452/459) and the control group (74%: 109/147; *p* < 0.001). The chromosomal anomaly rate was similar between the two groups: 52% (234/452) in the intervention and 42% (46/109) in the control group (*p* = 0.073; Table 1). The distribution of chromosomal anomalies in both groups was not significantly different (*χ*^2^ = 1,180; *p* = 0.09; Table 1). The anomalies most commonly found in the intervention group were trisomy 22 (*n* = 39), trisomy 16 (*n* = 37), and monosomy X (*n* = 33), accounting each for 14–17% of all the anomalies detected by QF-PCR. Additionally, there were 25 triploidies, nine double anomalies (five of them were a double trisomy), five monosomies 21, and five mosaicisms. Furthermore, QF-PCR was able to detect three complete uniparental disomies, in which pathology studies revealed a complete hydatidiform mole, and parental QF-PCR markers, a paternal origin of the extra haploid chromosomes set.

**Table 1:** Distribution of chromosomal anomalies revealed by the two rounds of QF-PCR in early pregnancy losses in the intervention and control groups.

Chromosomal anomalies	Intervention group		Control group	
	N	Frequency (%)	N	Frequency (%)
Trisomy 7	6	3	1	2
Trisomy 13	18	8	5	11
Trisomy 15	26	11	7	15
Trisomy 16	37	16	7	15
Trisomy 18	4	2	3	7
Trisomy 21	24	10	5	11
Trisomy 22	39	17	4	9
Monosomy 21	5	2	0	0
Monosomy X	33	14	7	15
Triploidy	25	11	4	9
Double anomaly	9	4	3	7
Mosaicism	5	2	0	0
Complete mole	3	1	0	0
Total	234	100	46	100

Among the 218 chorionic villi samples with normal results at the two rounds of QF-PCR (48%), there were 16 with an insufficient amount of DNA left (7%), and therefore 202 SNP-arrays were performed (Figure 1). SNP-array had one (0.5%) failed result and revealed 67 (33%) additional atypical chromosomal anomalies defined by a genomic imbalance greater than 10 Mb, and therefore detectable by karyotype. There were 50 atypical autosomal trisomies, six chromosomal rearrangements (all were *de novo* unbalanced anomalies: three reciprocal translocations, two deletions, and one tetrasomy 9p), 10 mosaicisms and one double anomaly (Table 2). Additionally, the SNP-array revealed seven (2.5%) pathogenic submicroscopic anomalies (microdeletions or microduplications), but no cases of uniparental disomies were found (Table 3). Among the seven CNVs identified, four were classified as pathogenic or likely pathogenic based on the gene content, its size, and/or the origin. The remaining two (1%) CNVs were considered VOUS, and thus, its role in the cause of pregnancy loss is difficult to predict. Among the 127 negative results at SNP-array, conventional karyotype revealed five non-mosaic tetraploidies, which may be causative of fetal demise, and go undetected by both QF-PCR and SNP-array.

**Table 2:** Distribution of chromosomal anomalies greater than 10 Mb found by the SNP-array in the early pregnancy losses with normal results at the two rounds of QF-PCR of the intervention group.

Chromosomal anomalies	N	%
Trisomy 2	4	6
Trisomy 4	4	6
Trisomy 6	1	1
Trisomy 8	4	6
Trisomy 9	6	9
Trisomy 10	9	13
Trisomy 11	3	4
Trisomy 12	4	6
Trisomy 14	6	9
Trisomy 17	3	4
Trisomy 20	6	9
Double anomaly	1	1
Chromosomal rearrangement	6	9
Mosaicism	10	15
Total	67	100

**Table 3:** Submicroscopic CNV identified by the SNP-array analysis in the early pregnancy losses with a normal karyotype of the intervention group.

Pathogenic copy number variant	Size	Gene content
arr[GRCh37] 7q11,23(71939063_77676448)x1	5.7 Mb	ELN, NCF1, POR, MDH2, HSPB1, YWHAG, ZP3, PTPN12
arr[GRCh37] 1q21,1q21,2(146334404_149202620)x1	2.8 Mb	GJA5, GJA8
arr[GRCh37] 6q23.1q23.2(130432067_133677770)x1	3.2 Mb	ARG1, MED23, ENPP1, VNN1, EYA4
arr[GRCh37] Xq26,3q27,1(134962013_138522776)x1	~3.5 Mb	FHL1, CD40LG, ARHGEF6, ZIC3, F9
arr[GRCh37] 19p13.11p12(19454548_23742080)x3	4.2 Mb	No OMIM genes associated with pathology
**VOUS**		
arr[GRCh37] Xp21,1(32030989_32077622)x1	4.6 Kb	DMD
arr[GRCh37] 4p16.3(2661045_2896952)x3 dn	0.2 Mb	SH3BP2

Taking into account the whole intervention group, the observed diagnostic yields were the following: 52% (234/452) for the two rounds of QF-PCR, and 17% (74/435) for the SNP-array, including 15% (67/435) for atypical chromosomal anomalies with a genomic imbalance greater than 10 Mb and 1.1% (5/435) for submicroscopic pathogenic variants. No differences were observed when recurrent (two or more losses; *n* = 97) and non-recurrent (*n* = 357) pregnancy losses were compared (Chi-square statistic = 0.3176; *p* = 0.989; Table 4).

**Table 4:** Distribution of results observed in recurrent (two or more losses) and non-recurrent pregnancy losses.

	Non-recurrent	Recurrent
Normal results	99 (27%)	28 (28%)
Trisomies 13,18,21, Monosmy X, Triploidy (First round QF-PCR)	99 (28%)	27 (28%)
Trisomy 15,16,22 (Second round QF-PCR)	87 (24%)	21 (22%)
Other chromosomal anomalies (>10 Mb)	52 (15%)	15 (15%)
Pathogenic copy number variants (<10 Mb)	4 (1%)	1 (1%)
Variants of unknown significance	2 (1%)	0 (0%)
Failed	14 (4%)	5 (5%)
Total	357 (100%)	97 (100%)

The mean maternal age was 35.8 years (range: 17–48 years), with significant differences between cases with normal (34.4 years) and abnormal results (36.2 years; *p* < 0.01). In a stratified analysis according to the type of genetic anomaly, maternal age was found to be significantly higher in pregnancy losses caused by autosomal trisomies (37.3 years), as compared to other anomalies (33.7 years; *p* < 0.001).

## Discussion

In this study, we assayed the implementation of three novel approaches in the genetic investigation of early pregnancy loss: (a) a stepwise molecular work-up based on two rounds of QF-PCR and SNP-array; (b) chorionic villi retrieval with the use of CVS before uterine evacuation, compared to a POC obtained at the surgical evacuation of the uterus content in the control group; and (c) a routine offer of genetic investigations from the first early pregnancy loss.

When comparing the methods of embryonic tissue retrieval between both groups, CVS (intervention group) was clearly superior to studying POC after uterine evacuation (control group), given that success rates were 98 and 79% (*p* < 0.001), respectively. Our group has shown that transcervical CVS is highly effective in obtaining samples in early pregnancy loss and that maternal contamination can be completely avoided with the use of the short-term culture (Soler et al., 2017). However, transcervical CVS is not common in Europe and requires a well-trained team. As far as we know, there is only one other group besides our team that has reported their results in early pregnancy loss with the use of CVS (Gimovsky et al., 2018). The drawbacks of karyotyping POC can certainly be overcome by SNP-arrays. Yet, the increasing use of medical management of retained POC, which is considered as safe as surgical management, is preventing its availability (American College of Obstetricians and Gynecologists' Committee on Practice Bulletins—Gynecology, 2018). In medical management, CVS performed previous to uterine content evacuation may become the elective method for obtaining fetal tissues. Cell-free DNA testing has been recently proposed by our group as an alternative when facing no availability of POC (Yaron et al., 2020).

Regarding the stepwise molecular work-up, the fact of restricting the SNP-array application to a half (218/454) of the studied samples with a normal QF-PCR result, in a similar manner to what is done in ongoing pregnancies, has the advantages of reducing costs and the turnaround time, as compared to the application of an SNP-array alone to the whole study population. This stepwise approach had only three diagnostic failures, and it is able to detect maternal cell contamination, triploidies, and complete hydatiform moles caused by a complete uniparental paternal disomy. A 52% diagnostic yield was observed for QF-PCR. As for the SNP-array, a 33% diagnostic yield was registered for atypical chromosomal anomalies and a 2.5% for submicroscopic pathogenic anomalies. Our work-up can be applied both in POC and in CVS, although one-fourth of POC samples was shown to be maternal instead of fetal. This finding is similar to the 22% reported rate of maternal contamination obtained from POC karyotyping (Lathi et al., 2014). A protocol combining QF-PCR and CMA was first described by Wou et al. (2016) and has also been introduced in two other leading centers in London (Donaghue et al., 2017) and Naijing (Wang et al., 2020). Our novel contribution was to add a second QF-PCR round with chromosomes 15, 16, and 22. London’s large series of recurrent pregnancy losses showed that the combined use of QF-PCR and comparative genomic hybridization array (CGH-array) in POC provides a better cost efficiency than testing by array-CGH alone. This series showed a 1.4% failure rate, which is similar to the 0.6% rate found in our study.

Chromosomal Microarray Analysis is nowadays considered the best method for POC analysis. Hence, a recent meta-analysis from our group showed that when CMA is performed instead of a karyotype in early pregnancy loss, the success rate increases 27 percentage points, from 68 to 95% (Pauta et al., 2018). The second advantage of CMA is the incremental yield of CMA above the karyotype, because it enables the search for submicroscopic anomalies (from 10 Mb to 10–100 Kb in size), which account for 2% of the cases according to our recent meta-analysis (Pauta et al., 2018). If the present series would be added to those previously included in a meta-analysis from our own group, six recurrent CNVs associated to miscarriage would be identified: del1q21.1, del1p36.33, del3p26, del7q11.23, dup11p15.5, and del22p13. Among those, only the 7q11.23 microdeletion has been previously described as probably to be associated with miscarriage (Wang et al., 2017). In the present series, a 0.3% (1/308) prevalence for the 7q11.23 microdeletion, causative of Williams-Beuren syndrome, was observed, accounting for a 0.3% (1/308) of miscarriages and overlapping the previously reported range in early pregnancy losses (0.05–0.2%; Levy et al., 2014; Wang et al., 2017), much higher than that in the general population (0.013%; Merla et al., 2010). A recent systematic review by Wang et al. (2020) found nine CNVs associated to miscarriage, and one of those, del7q11.23, has also been found in our series. A second microdeletion, del1q21.1, has been identified in the previous studies (Levy et al., 2014; Sahoo et al., 2017), but it is not a so-well-established cause of miscarriage.

Finally, we wish to point out that our routine offer to women with early pregnancy loss had a wide acceptance since uptake was 82% (459/559) during the study period. Clinical guidelines only consider recurrent pregnancy loss as a reason to undertake any diagnostic work-up. In an opinion commentary, we argued that in early pregnancy loss, similarly to stillbirth, women commonly wish to know the cause of the loss and its recurrence risk (Borrell and Stergiotou, 2013). Genetic testing is recommended in stillbirths even though it may only provide a clear result in 5–10% of the cases. It is intriguing that contrary, this testing is not recommended in early pregnancy loss although it could reveal the cause of 2/3 of the cases. Our experience shows that, when offered, 82% of women preferred to undergo a CVS before any surgical or medical treatment in order to know the reason of the loss. A recent meta-analysis has demonstrated that significant depression (25%) and anxiety (40% similar to termination of pregnancy) in the first month follow early pregnancy loss in women. There is also evidence of post-traumatic stress symptoms (30%) in three studies (Farren et al., 2018). Furthermore, it has been shown that identifying the cause of the loss help reduce the feelings of self-blame (Nikcevic et al., 1999) and has a major impact on the couple’s future reproductive plans (Borrell and Stergiotou, 2013). Although it is not recommended by the clinical guidelines, the fact that there are large series reporting the use of arrays in POC of non-recurrent early pregnancy losses supports this rationale (Levy et al., 2014; Wang et al., 2014, 2017; Wou et al., 2016; Sahoo et al., 2017; Qu et al., 2019).

One of the strengths of our study is the accuracy of the cytogenetic diagnosis, since both short- and long-term culture karyotypes were performed concurrently of the proposed new work-up based on two rounds of QF-PCR and SNP-array. Some other highlights of our study include the reliability of the results due to the homogenous molecular/cytogenetic procedures carried out in a single laboratory and the high success rate of QF-PCR along with SNP-array and conventional karyotyping. A major limitation of our study is that, in the control group of POC, cases were enrolled in another site and those with a normal QF-PCR in this group did not undergo SNP-array analysis due to limited funding. Another limitation is the lack of cost-effectiveness analysis in non-recurrent losses, when the current cost of its psychological impact is hard to be estimated, and it is out of the scope of the present study. We are also aware that offering molecular testing to all women with an early pregnancy loss brings further need for funding genetic counseling, which can be helpful in overcoming loss and in planning of their reproductive future, in addition to the cost of CVS and molecular tests. Further limitations of the present study are the limited number of cases studied and the exclusion of complete miscarriages.

In summary, we propose to offer a new stepwise molecular work-up to diagnose chromosomal abnormalities to those women who have had one or more early pregnancy losses, even if this was their first event. Chorionic villi may be retrieved during the surgical treatment or before medical or expectant management by means of transcervical CVS. Our results show that if karyotyping is replaced by SNP-array in QF-PCR normal cases, further 33% (67/202) chromosomal anomalies and 2.5% (5/202) submicroscopic anomalies will be revealed. We are aware that the feasibility of testing all women that have experienced an early pregnancy loss is questionable, and therefore, we would suggest that it would be offered at least for women who suffer from recurrent pregnancy loss.

## Data Availability Statement

The data analyzed in this study is subject to the following licenses/restrictions: The data that support the findings are available for the editor. The data are not publicly available due to privacy and ethical restrictions. Requests to access these datasets should be directed to mpauta@clinic.cat.

## Ethics Statement

The studies involving human participants were reviewed and approved by IRB of Hospital Clinic de Barcelona (HCB/2014/6020), and written informed consent was obtained from all women. The patients/participants provided their written informed consent to participate in this study.

## Author Contributions

CB carried out the QF-PCR. LR-R carried out the CMA and AS carried out the conventional Karyotype. They contributed to the interpretation of the results. MG processed the experiment data and created the database. JS included the cohort of patients from HSJD and VB and AB included the cohort from HCB. They supervised the inclusion of patients and provided the samples after the patient signed CI. CI took care of patients and sample handling. MP processed the experimental data, performed the analysis, drafted the manuscript, and designed the Figures. AB designed the project, supervised the research, and co-wrote the manuscript. All authors contributed to the article and approved the submitted version.

### Conflict of Interest

The authors declare that the research was conducted in the absence of any commercial or financial relationships that could be construed as a potential conflict of interest.
